# Clinical Features and Outcome in Adult Cases of Tuberculous Meningitis in Tertiary Care Hospital in Antananarivo, Madagascar

**DOI:** 10.1155/2017/9316589

**Published:** 2017-03-15

**Authors:** Mihaja Raberahona, Rivonirina Andry Rakotoarivelo, Tiana Razafinambinintsoa, Radonirina Lazasoa Andrianasolo, Mamy Jean de Dieu Randria

**Affiliations:** ^1^Service des Maladies Infectieuses, Hôpital Universitaire Joseph Raseta Befelatanana, Antananarivo, Madagascar; ^2^Service des Maladies Infectieuses, CHU Tambohobe, Fianarantsoa, Madagascar; ^3^Université de Fianarantsoa, Fianarantsoa, Madagascar; ^4^Programme National de Lutte contre la Tuberculose (PNLT), Ministère de la Santé Publique, Antananarivo, Madagascar

## Abstract

*Purpose.* We aimed to describe and to assess prognosis factors in tuberculous meningitis in adult patients.* Methods.* We performed a retrospective study of case records of adult patients. Patients classified as definite, probable, or possible tuberculous meningitis according to standardized definition criteria were included and assessed in the study.* Results.* Seventy-five patients were included in the study. Tuberculous meningitis was classified as definite in 8 (10.7%), probable in 44 (58.7%), and possible in 23 patients (30.6%). HIV was found in 3% of patients. Patients were in advanced stages at admission in 82.7%. Median duration of symptoms prior to admission was 3 weeks (IQR: 2–5). Median time to diagnosis following admission was 5 days (IQR: 3–8). Median CSF WCC was 75 per mm^3^ with lymphocytic predominance in 38 cases (52.8%). Median CSF glucose level was 1.48 mmol/L and median CSF protein level was 1 g/L. Mortality rate was 28%. Age ≥ 35 years (aOR: 4.06; 95% CI: 1.16–14.26) and coma (aOR: 12.98; 95% CI: 1.13–149.16) predicted inpatient mortality.* Conclusion.* Most of the patients experienced more than 3 weeks of diagnostic delay prior to admission. Mortality was high and occurred early after admission. Age and coma were identified as independent prognosis factors.

## 1. Introduction

Madagascar ranks among countries where tuberculosis is highly endemic. In 2013, the World Health Organization (WHO) estimated the prevalence of tuberculosis to be 442 per 100,000 population (95% CI: 222–735) and incidence to be 234 per 100,000 population (95% CI: 139–280). Estimated mortality rate was 46 per 100,000 population (95% CI: 19–84). A total of 19,010 new cases of pulmonary tuberculosis and 4,964 new cases of extrapulmonary tuberculosis were notified [1]. Tuberculous meningitis (TBM) accounts for approximately 1% of all forms of tuberculosis and 5.1% of extrapulmonary tuberculosis [2, 3]. However, TBM is associated with both high rate of mortality and morbidity. Indeed, mortality rate ranges between 40.3% and 87.9% in Africa and neurological sequelae may affect up to 78.5% of surviving patients during follow-up [4, 5]. Moreover, the diagnosis of TBM is difficult due to nonspecific clinical presentation and lack of sensitivity of microbiological confirmation tests [6]. In addition, in the context of resource-limited countries as Madagascar with weak health system and limited access to laboratory tests, diagnosis of TBM remains a challenge. Thereby, TBM is probably under diagnosed and not well known in Madagascar.

In this study, we aimed (i) to describe clinical, laboratory, and radiological findings and (ii) to assess prognosis factor in adult patients with TBM.

## 2. Methods

### 2.1. Study Setting and Design

We conducted this single-center study in the Infectious Diseases Unit of the University Hospital Joseph Raseta Befelatanana, Antananarivo, Madagascar. Antananarivo is the capital and the largest city of Madagascar. The University Hospital Joseph Raseta Befelatanana is a tertiary care reference hospital dedicated to medical specialties with approximately 350 beds. About 60 and 80 patients are admitted monthly in the Infectious Diseases Unit. We retrospectively reviewed the case records of adult patients (age ≥ 16 years) with TBM admitted between 2007 and 2014.

### 2.2. Case Definition

Patients classified as definite, probable, or possible TBM according to standardized case definition proposed by Marais et al. [7] were included in this study. Thereby, patients were considered as having a definite TBM if evidence of* Mycobacterium tuberculosis* (MTB) is found in the cerebrospinal fluid (CSF) either by detection of acid-fast bacilli (AFB) on microscopy or by culture or by molecular technique such as nucleic acid amplification (NAA) test. The definition of probable or possible TBM depends on diagnostic score based on clinical, CSF, and radiological criteria. A maximum of 6 points is allocated to clinical criteria which includes the following items: symptom duration of more than 5 days (4 points), systemic symptoms suggestive of tuberculosis (2 points), history of recent close contact with an individual with pulmonary tuberculosis or a positive tuberculosis skin test or interferon-gamma release assay (2 points), focal neurological deficit (1 point), cranial nerve palsy (1 point), and altered consciousness (1 point). A maximum of 4 points is allocated to CSF criteria including CSF clear appearance, cells count between 10 and 500 per *µ*l, lymphocytic predominance (>50%), protein level greater than 1 g/L, and CSF to plasma glucose ratio of less than 50% or an absolute CSF glucose concentration less than 2 mmol/L. Each item corresponds to 1 point. A maximum of 6 points is allocated to cerebral imaging criteria which includes the following items: hydrocephalus (1 point), basal meningeal enhancement (2 points), tuberculoma (2 points), infarct (1 point), and precontrast basal hyperdensity (1 point). A maximum of 4 points is allocated to evidence of tuberculosis elsewhere criteria which include the following items: chest radiograph suggestive of active tuberculosis (2 points of signs of tuberculosis and 4 points if miliary tuberculosis), evidence of tuberculosis outside the central nervous system on computed tomography, MRI, or ultrasound (2 points), evidence of MTB by detection of AFB or by culture from extraneural specimen (4 points), and positive nucleic acid amplification test for MTB from extraneural specimen (4 points). Patients with total score ≥10 points (when cerebral imaging is not available) or ≥12 points (when cerebral imaging is available) were considered as having a probable TBM. Patients with total score between 6 and 9 points (when cerebral imaging is not available) or score between 6 and 11 points (when cerebral imaging is available) were considered as having possible TBM.

### 2.3. Data Collection

Demographic, clinical, laboratory, and imaging data were collected from patient records. Clinical data include clinical signs at presentation. The duration of symptoms is defined as the time elapsed between the onset of signs and hospitalization. The time to diagnosis is the duration between admission to hospital and the start of antituberculosis therapy. Clinical severity at admission was assessed by using the British Medical Research Council (BMC) staging for TBM [8]. Grade 1 corresponds to alert and oriented patient without focal neurological deficits. Grade 2 is defined as a Glasgow coma scale of 11 to 14 or 15 with focal neurological deficits. Grade 3 is defined as a Glasgow coma scale of 10 or less with or without focal neurological deficits. Thwaites' diagnostic index (TDI) [9] was assessed for each case. This scoring system allows differentiating TB meningitis from bacterial meningitis. TDI is obtained by adding the score for age (0 if age < 36 years and +2 if age ≥ 36 years), for length of history (0 if <6 days and −5 if ≥6 days), for peripheral blood white cells count (WCC) (0 if <15,000 per mm^3^ and +4 if ≥15,000 per mm^3^), for CSF total WCC (0 if <900 per mm^3^ and +3 if ≥900 per mm^3^), and for CSF neutrophils percentage (0 if <75% and +4 if ≥75%). A total score ≤ 4 predicts the diagnosis of TBM and a total score > 4 predicts the diagnosis of bacterial meningitis with a sensitivity of 86% and a specificity of 79%. Laboratory data include CSF and other microbiological findings (sputum and gastric aspirate smear for AFB). Radiological findings include chest X-ray and cerebral imaging when available.

### 2.4. Statistical Analysis

Categorical variables were compared using Chi-square test or Fischer's exact test where appropriate. Predictors of mortality were assessed using logistic regression analysis. Univariate analysis was done to identify variables associated with mortality. Thereby, variables identified in univariate analysis with *p* value <0.2 were entered into a multivariate logistic regression model to identify independent mortality risk factors. A *p* value <0.05 was considered as significant. Statistical analyses were performed using Stata 12.0 (StataCorp LP, Texas, USA).

## 3. Results

### 3.1. Clinical Findings

A total of 75 patients were included in this study. Among them, 8 patients (10.7%) had definite TBM, 44 (58.7%) had probable TBM, and 23 (30.6%) had possible TBM. The mean age was 35.4 ± 12.7 years (range 16–71 years). Forty-two patients (56%) were male and 33 (44%) were female. All patients were screened for HIV and only 3 patients (4%) were HIV positive. Five patients (6.7%) had tuberculosis history including 3 pulmonary tuberculosis and 2 extrapulmonary tuberculosis. Three patients (4%) reported close contact with person with active tuberculosis. Twelve patients (16%) had received BCG vaccination. Median duration of symptoms prior to admission was 3 weeks (IQR: 2–5). Duration of symptoms was < 1 week for 2 patients (2.7%), between 1 and 4 weeks for 37 patients (49.3%), and ≥ 4 weeks for 36 patients (48%). Median time to diagnosis was 5 days (IQR: 3–8). Time to diagnosis was < 3 days for 13 patients (17.3%), between 3 and 7 days for 38 patients (50.7%), and ≥ 7 days for 24 patients (32%). TDI could be assessed for 68 patients. All patients had a score consistent with the diagnosis of TBM according to TDI (score ≤ 4). Among them, 29 (42.6%) had TDI of −5 (maximal score for the diagnosis of TBM according to TDI). Clinical findings on admission are detailed in Table 1.

### 3.2. CSF Findings

CSF had clear appearance in all cases. Median CSF WCC was 75 per mm^3^ (IQR: 29–175). However, CSF WCC was < 10 per mm^3^ in 8 cases (10.7%). CSF lymphocytes percentage could be assessed in 72 cases. It was < 25% in 8 cases (11.1%), between 25% and 50% in 15 cases (20.8%), and between 50% and 75% in 11 cases (15.3%). CSF lymphocyte predominance (≥75%) was found in 38 cases (52.8%). CSF glucose level was available for 74 patients. Median CSF glucose level was 1.48 mmol/L (IQR: 0.76–2.55). CSF glucose level was < 1.1 mmol/L in 26 cases (35.1%), between 1.1 and 2.2 mmol/L in 26 cases (35.1%), and ≥ 2.2 mmol/L in 22 cases (29.8%). CSF protein level was available for 74 patients. Median CSF protein level was 1 g/L (IQR: 0.5–2.4). CSF protein level was normal (<0.5 g/L) in 16 cases (21.6%), between 0.5 and 1 g/L in 11 cases (14.9%), and ≥ 1 g/L in 47 cases (63.5%).

### 3.3. Radiological Findings

Sixty-eight patients (90.7%) had chest X-ray. Chest radiological exam was abnormal in 43 cases (63.2%). Major abnormalities were parenchymal infiltration (*n* = 25, 36.8%), miliary pattern (*n* = 18, 26.5%), pleural effusion (*n* = 4, 5.9%), and cavitary lesions (*n* = 1, 1.5%). Fifteen patients (20%) had brain computed tomography scan. Seven patients (46.7%) had an abnormal finding. Major findings were hydrocephalus (*n* = 5, 71.4%) and basal enhancement (*n* = 3, 60%).

### 3.4. Microbiological Findings

Among the 75 patients, only 13 patients (17.3%) had a microbiological examination of the CSF for MTB identification. MTB were identified in the CSF samples of 8 patients. CSF culture was positive in 3 patients. NAA test for MTB from CSF sample was positive for one patient. CSF culture and NAA test for MTB were both positive in 4 patients. AFB were identified from sputum in 9 patients (12%).

### 3.5. Outcome

Antituberculosis therapy was started after diagnosis of TBM according to national guidelines. All patients except 5 patients who had tuberculosis history received the first-line regimen including rifampicin, isoniazid, ethambutol, and pyrazinamide during the intensive phase of the treatment (2 months). These four drugs were administered by oral route. Before 2012, patients received ethambutol and isoniazid for 6 months during the continuation phase of the treatment. After 2012, patients received rifampicin and isoniazid for 4 months during the continuation phase of the treatment. The 5 patients with tuberculosis history received rifampicin, isoniazid, ethambutol, pyrazinamide, and streptomycin during 2 months followed by continuation phase including rifampicin, isoniazid, ethambutol, and pyrazinamide.

Twenty-one (28%) out of the 75 patients died. Median time to death was 8 days (IQR: 6–15) after admission to the hospital.  Table 2 details univariate analysis of variables associated with inpatient mortality. Factors associated with inpatient mortality were age ≥ 35 years (*p* = 0.044) and coma (*p* = 0.048). In multivariate analysis by logistic regression model (*p* = 0.0109, *R*^2^ = 0.22), age ≥35 years (adjusted odds ratio [aOR] 4.06, 95% confidence interval [95% CI] 1.16–14.26, *p* = 0.029) and coma (aOR 12.98, 95% CI 1.13–149.16, *p* = 0.040) predicted inpatient mortality (Table 3).

## 4. Discussion

TBM remains an extremely serious disease. Most of the patients in our study (82.7%) presented with advanced clinical stage (BMC grades 2 and 3). Several studies had reported similar observations [10, 11]. Moreover, we found that median duration of symptoms prior to admission was 3 weeks and only 2 patients out of 75 had less than a week of duration of symptoms. Duration of symptom prior to admission reported in previous studies was commonly longer than one week which is consistent with a subacute meningitis [12–14]. However, duration of symptoms prior to admission in our study was substantially longer than reported in these studies. Apart from these clinical features, the lack of health coverage and the weakness of the healthcare system lead patients to delay their admission due to the high cost of hospitalization which must be fully assumed by the patients themselves. This can partially explain the high proportion of patients who presented with advanced stage in our study.

TBM often occurred in patients without predisposing conditions in our study. Only 3 patients had HIV infection and 5 patients had history of tuberculosis. However, in African countries, rate of HIV infected patients among those with TBM is extremely high. Marais et al. [15] reported 88% of HIV infected patients among patients with TBM in African setting. This difference may be related to low prevalence of HIV in Madagascar. Indeed, the World Health Organization estimated HIV prevalence to 264 per 100,000 population in 2014 [16]. Moreover, in high income countries with low prevalence of tuberculosis, TBM as well as tuberculosis in general often occur in patients coming from high prevalence countries [13, 17, 18]. Apart from HIV infection, other predisposing conditions including tuberculosis history, intravenous drug use, diabetes mellitus, alcoholism, or various host immunosuppression factors were commonly found in studies conducted in high income countries [19, 20]. The profile of patients with TBM in our study is probably influenced by high burden of tuberculosis contrasting with low prevalence of HIV. Thereby, TBM tends to occur in patients without predisposing factors. However, we noted that only 16% of the patients had received BCG vaccination. Although the protective role of BCG vaccination against TBM in children is well known [21], its effect in adults remains uncertain [22]. However, Aronson et al. [23] and recently Nguipdop-Djomo et al. [24] demonstrated long-term efficacy of BCG vaccination against tuberculosis in general. Nevertheless, the proportion of vaccinated patients among those with TBM is currently low in setting applying the same strategy for BCG vaccine as in Madagascar [5, 12].

In resource-constrained setting, as in Madagascar, diagnosis of TBM remains a challenge which considerably contributes to diagnostic delay and underdiagnosis. In fact, CSF microbiological examination for MTB including detection of AFB, culture, or NAA test were only done for 13 patients (17.3%) due to intermittent access to these diagnostic tests. In all cases, the diagnosis of TBM was based on clinical features and CSF findings. Indeed, these features could help to differentiate TBM from other etiology of meningitis. Previous study demonstrated that clinical features including duration of symptoms > 5 days, presence of headache, and CSF findings including CSF WCC < 1000 per mm^3^, CSF clear appearance, CSF lymphocytes > 30%, and CSF protein level help to distinguish TBM from acute bacterial meningitis with sensitivity of 93% and specificity of 77% when three or more of these features are present [25]. TDI allowed differentiating bacterial meningitis from TBM based on 5 criteria weighted by a score including age, duration of symptoms, peripheral blood WCC, CSF WCC, and CSF percentage of neutrophils [9]. Assessment of TDI in different studies including non-HIV adults patients showed sensitivity from 95.6% to 99% and a specificity from 70.8% to 80% [26, 27]. However, specificity is too low when test was applied in high HIV prevalence setting [28]. We retrospectively applied this diagnostic index in patients included in this study. All patients had a score consistent with the diagnosis of TBM. TDI could be useful in diagnosing TBM in our setting with low HIV prevalence and probably need further assessment.

In our study, patients received antituberculosis therapy following national guidelines and received 8-month or 6-month regimen. All forms of tuberculosis including TBM are treated with the same regimen and duration. However, most of international guidelines including current WHO guideline recommend longer course of treatment ranging from 9 to 12 months [29–31]. Furthermore, short-term therapy is not supported by strong evidence [32]. However, neither the length nor the optimal treatment regimen is well known in TBM.

Overall mortality in our study was 28%. Mortality rate in our setting is considerably lower than reported in other African countries where mortality ranged from 59.9% to 87.9% [4]. This difference is likely due to lower HIV prevalence in our setting as HIV infection is a major prognosis factor among TBM patients [33]. However, our study has important limitation due to its retrospective nature which may contribute to underestimating long-term mortality and sequelae. In fact, patient follow-up is not always possible. After discharge from hospital, patients are referred to primary care setting near to their home to promote adherence to antituberculosis therapy. In our study, age ≥ 35 was identified as an independent predictor of mortality. Previous studies also found that older patients had higher risk of death compared to younger patients with different threshold compared to our finding [34–36]. This difference is likely due to age distribution. Nevertheless, Shaw et al. [37] demonstrated a linear relationship between age and risk of death. Apart from age, coma was also found to be an independent risk of death in our study. Several studies reported similar observations [34, 38]. In our study, death occurred within 8 days following admission. Although we failed to demonstrate relationship between treatment delay and mortality, previous study showed that patients receiving antituberculosis therapy within 1 day of initial presentation had better prognosis compared with patients receiving antituberculosis therapy after 1 day. Likewise, patients who started antituberculosis therapy immediately or after nonspecific antibiotics within 5 days had better prognosis [39]. In our setting, most of the patients received empiric antimicrobial therapy before antituberculosis therapy since lack of clinical improvement with nonspecific antimicrobial therapy is considered as an evidence for the diagnosis of TBM. Indeed, time to diagnosis was ≥ 3 days in most of the cases. However, this approach tends to postpone initiation of appropriate antituberculosis therapy.

Several limitations were identified due to the retrospective design of this study. We were unable to assess long-term prognosis of patients. Some biological data such as blood glucose were not always available and did not allow assessing CSF to blood glucose ratio for the majority of the patients. CSF microbiological examination for MTB as well as cerebral imaging was limited and not routinely performed because of its cost which was entirely borne by the patient. However, this retrospective study allowed describing what was done in daily clinical practice.

## 5. Conclusion

In this study, most of patients with TBM presented with advanced stages at admission. HIV infection rate as well as BCG vaccination rate among TBM patients remained low. Clinical features were mostly consistent with subacute meningitis. CSF examination showed moderate pleocytosis with lymphocytic predominance, low glucose level, and increased protein level in the majority of cases. Microbiological confirmation of TBM was low due to poor access to culture or molecular test for MTB identification. Mortality rate was 28% and death generally occurred early after admission. Age ≥ 35 years and coma were identified as independent predictors of mortality. In resource-constrained settings, the diagnosis of TBM should be based promptly on clinical findings and CSF features as microbiological confirmation test are not always available. Moreover, antituberculosis therapy should be started immediately whenever clinical and CSF findings are consistent with the diagnosis of TBM to improve outcome.

## Figures and Tables

**Table 1:** Clinical findings on admission in patients with TBM.

Clinical findings	*n* (%)
Fever	72 (96.0)
Headache	58 (77.3)
Weight loss	33 (44.0)
Persistent cough (≥2 weeks)	22 (29.3)
Photophobia	7 (9.3)
Seizures	19 (25.3)
Neck stiffness	52 (69.3)
Altered consciousness	55 (73.3)
Coma	6 (8.0)
Cranial nerve palsy	13 (17.3)
Focal neurological signs	8 (10.7)
BMC	
Grade 1	13 (17.3)
Grade 2	54 (72.0)
Grade 3	8 (10.7)

**Table 2:** Univariate analysis of variables associated with inpatient mortality in TBM.

Variable	Survived	Died	*p* value
	*n* (%)	*n* (%)	
	54 (72.0)	21 (28.0)	
Age ≥ 35 years	22 (40.7)	14 (66.7)	0.044
Male gender	28 (51.8)	14 (66.7)	0.246
HIV infected	2 (3.7)	1 (4.8)	0.633
Symptoms prior to admission ≥ 4 weeks	24 (44.4)	12 (57.1)	0.323
Time to diagnosis ≥ 7 days	18 (33.3)	6 (28.6)	0.691
BCG vaccination	11 (20.4)	1 (4.8)	0.090
TB history	3 (5.6)	2 (9.5)	0.432
Weight loss	21 (38.9)	12 (57.1)	0.153
Cough ≥ 2 weeks	16 (29.6)	6 (28.6)	0.928
Photophobia	5 (9.3)	2 (9.5)	0.972
Seizures	16 (29.6)	3 (14.3)	0.140
Neck stiffness	37 (68.5)	15 (71.4)	0.806
Altered consciousness	41 (75.9)	14 (66.7)	0.416
Coma	2 (3.7)	4 (19.1)	0.048
Cranial nerve palsy	11 (20.4)	2 (9.5)	0.224
Focal neurological signs	6 (11.1)	2 (9.5)	0.604
CSF WCC ≥ 150 per mm^3^	16 (29.6)	11 (52.4)	0.065
CSF glucose level ≥ 1.1 mmol/L	37 (69.8)	11 (52.4)	0.157
CSF protein level ≥ 1 g/L	31 (58.5)	16 (76.2)	0.154
Active pulmonary TB	5 (9.3)	4 (19.1)	0.241

**Table 3:** Multivariate analysis of factors associated with inpatient mortality.

Variable	aOR	95% CI	*p* value
Age ≥ 35 years	4.06	1.16–14.26	0.029
BCG vaccination	0.19	0.02–1.99	0.165
Weight loss	2.43	0.65–9.11	0.187
Seizures	0.35	0.06–1.98	0.233
Coma	12.98	1.13–149.16	0.040
CSF WCC ≥ 150 per mm^3^	2.31	0.61–8.68	0.217
CSF protein level ≥ 1 g/L	2.84	0.72–11.14	0.135
CSF glucose level ≥ 1.1 mmol/L	1.72	0.38–7.69	0.478
